# One-Stage Technique with Calcaneal Graft for the Treatment of Brachymetatarsia: A Case Report

**DOI:** 10.3390/medicina61030497

**Published:** 2025-03-13

**Authors:** Mercedes Ortiz-Romero, Álvaro Fernández-Garzón, Rocío Caceres-Matos, Raquel García de la Peña, Ana M. Rayo-Perez, Luis M. Gordillo-Fernández

**Affiliations:** 1 Department of Podiatry, Faculty of Nursing, Physiotherapy and Podiatry, University of Seville, 41009 Seville, Spain; mortiz17@us.es (M.O.-R.); raquelgp@us.es (R.G.d.l.P.); dra.anarayo@gmail.com (A.M.R.-P.); lgordillo@us.es (L.M.G.-F.); 2 Private Practice in Podocorp Clinic, 11201 Algeciras, Spain; 3 Nursing Department, Faculty of Nursing, Physiotherapy and Podiatry, University of Seville, Research Group CTS-1050: Complex Care, Chronicity and Health Outcomes, 41009 Seville, Spain; rcaceres3@us.es

**Keywords:** brachymetatarsia, metatarsal lengthening, chronic metatarsalgia, structural bone graft, reconstructive foot surgery, biomechanical alterations, postoperative functional outcomes

## Abstract

Brachymetatarsia is a rare congenital anomaly characterized by the shortening of one or more metatarsals, which can lead to functional impairment, pain, and aesthetic concerns. This case report describes a 17-year-old female patient with brachymetatarsia affecting the third and fourth metatarsals of the right foot, which was unresponsive to conservative treatment and caused persistent pain while standing. To address this condition, a single-stage surgical approach was performed using an autologous calcaneal bone graft to lengthen the affected metatarsals. Additionally, the second and fifth metatarsals were shortened to restore a physiological metatarsal parabola and resolve chronic metatarsalgia. The procedure resulted in complete correction of the metatarsal parabola, full resolution of metatarsal pain, and satisfactory functional recovery. The use of an autologous calcaneal graft proved to be an effective and reliable surgical option due to its cortico-cancellous composition, high osteogenic potential, and low antigenicity. This case highlights the advantages of autologous bone grafting as a valuable technique in the surgical management of brachymetatarsia.

## 1. Introduction

Brachymetatarsia is a congenital deformity characterized by abnormal shortening of one or more metatarsals due to premature closure of the growth plate [1]. This condition primarily affects the fourth metatarsal, followed by the first, with a marked predominance in females (14.8:1) and a bilateral prevalence in approximately 25.8% of cases [2,3]. Although some patients remain asymptomatic, the deformity can cause transfer pain, metatarsalgia, calluses, soft tissue contractures, and cosmetic concerns, especially during adolescence [4,5].

The diagnosis of brachymetatarsia is based on weight-bearing radiographs, where the affected metatarsal is found to be more than 5 mm proximal to the normal metatarsal parabola as described by Lelievre [6]. In addition, it may be associated with secondary deformities such as claw or hammertoes, and, in severe cases, with significant functional impairment of the forefoot [7,8].

Conservative management, including the use of orthoses and specialized footwear, can be helpful in relieving pain and improving forefoot pressure distribution. However, these strategies do not correct the underlying deformity, and in symptomatic cases or those with significant cosmetic implications, surgery is used to restore metatarsal length and foot function [9,10].

Among the surgical options, bone lengthening can be performed using two main approaches: acute single-stage lengthening and gradual distraction. The former, which includes techniques such as the interposition of autologous or allogeneic bone grafts, is a faster option and has lower complication rates but is limited in the amount of lengthening that can be achieved [11,12]. On the other hand, gradual distraction allows greater bone elongation but requires prolonged use of external fixators and is associated with a higher risk of complications, such as infections, fractures and joint stiffness [13,14]. Bone grafts, particularly autologous bone grafts, have been shown to be effective in one-stage procedures for moderate deformities, providing stability and good cosmetic and functional results [4,15].

Despite advances in surgical techniques, there is no definitive consensus on the best option for treating brachymetatarsia. Treatment selection should be based on patient-specific characteristics, such as desired length, age, aesthetic expectations, and risk of complications [16]. In this context, the present manuscript reports a clinical case of brachymetatarsia treated by single-stage lengthening with autologous bone grafting, highlighting the clinical and functional results obtained.

## 2. Case Report

We present the case of a 17-year-old female patient who presented herself for consultation with severe pain in the central metatarsals of her right foot. The patient´s informed consent form has been submitted. This is a young patient with a medical history without systemic, cardiac, neurological, or rheumatological diseases.

Clinical examination revealed a valgus foot with hyperkeratosis under the third and fourth metatarsal heads. The patient had been wearing unloading plantar orthoses for 8 years. Conservative treatment had not achieved its objective, and the patient was living with continuous pain EVA 8 caused by metatarsalgia at the age of 17 years.

**Figure 1:**
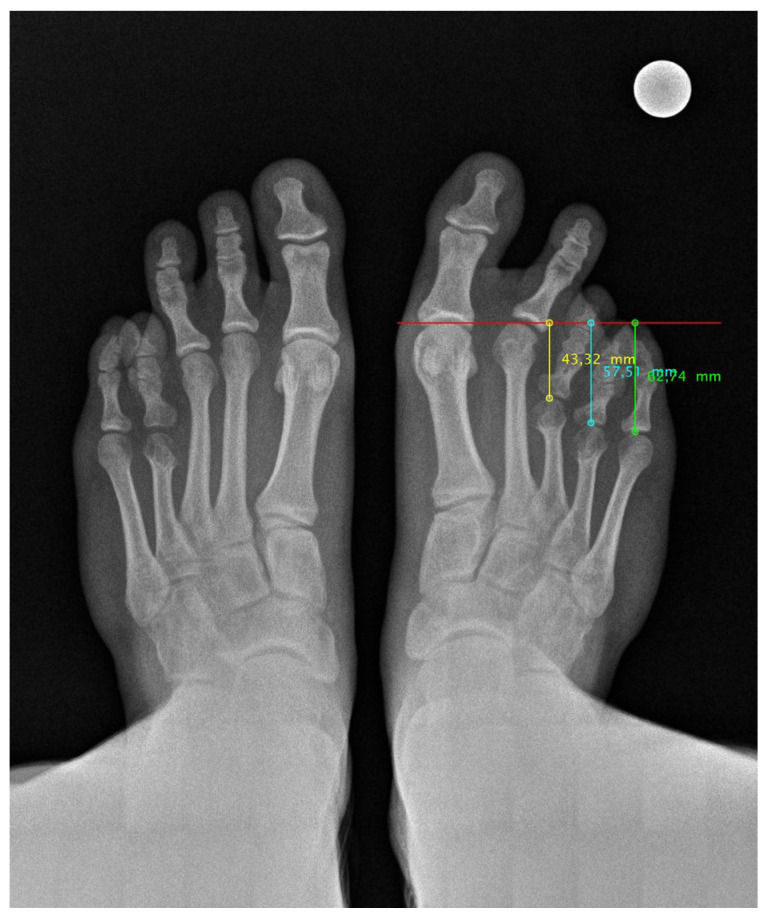
Pre-surgical radiological image of both feet under load.

Finally, the radiological study confirmed a diaphyseal shortening of the third and fourth radius of the right foot (Figure 1). Her left foot also showed diaphyseal shortening of only the fourth radius with no symptoms.

### 2.1. Surgical Procedure

The patient received a spinal block with 0.75% bupivacaine, along with sedation administered by the anesthesiology team. A tourniquet was placed 10 cm below the fibular head with a pressure of 250 mmHg.

#### 2.1.1. Bone Graft Harvesting

To facilitate access to the lateral aspect of the calcaneus, the patient’s leg was placed in internal rotation. A slightly oblique incision was made 2 cm above the calcaneus and parallel to the Achilles tendon. Careful blunt dissection was performed to identify and protect the sural nerve and lateral calcaneal branch.

**Figure 2:**
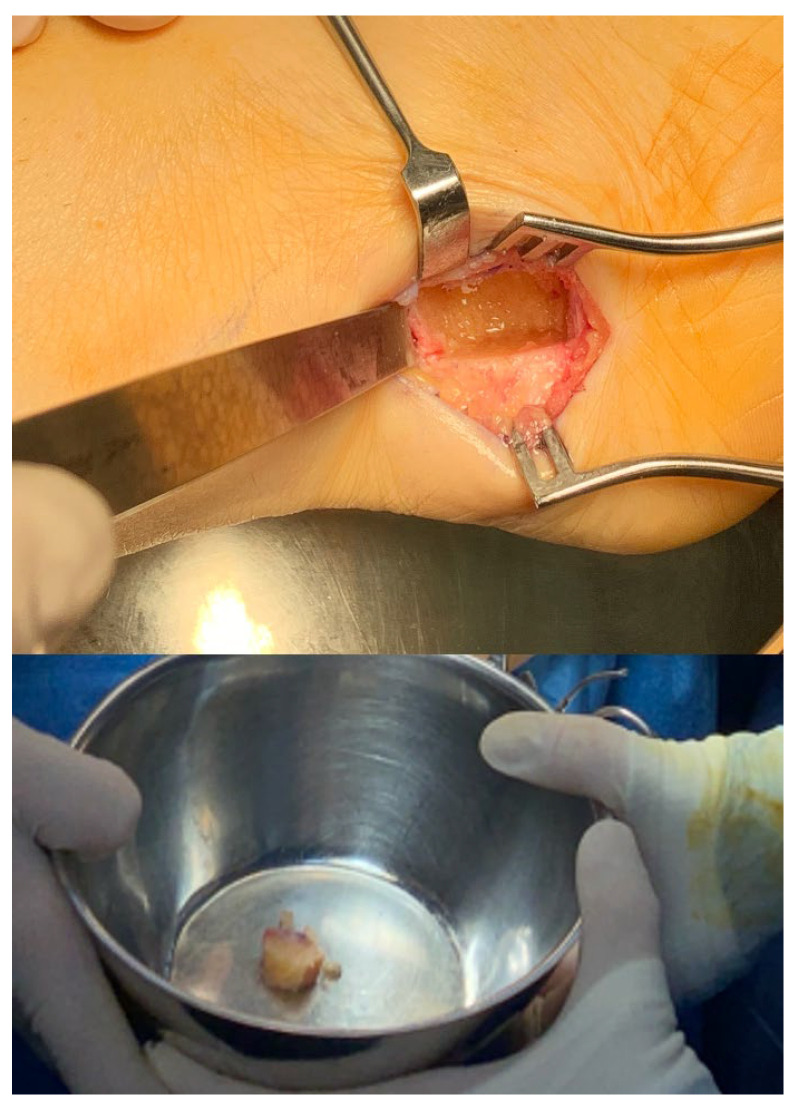
The appearance of the calcaneus after bone graft extraction can be observed, utilizing the cancellous bone of the calcaneus while preserving the medial cortical bone.

Once bone exposure was achieved, a ruler was used to measure and mark the desired graft size (1.8 cm × 1.1 cm) on the bone surface with electrocautery. Using a sagittal saw, the outer cortical bone was cut, and the graft was carefully extracted with an osteotome and mallet, ensuring preservation of the medial cortical bone to maintain structural integrity (Figure 2). The extracted graft was then shaped to match the recipient site, resulting in an implanted graft measuring 1.7 cm × 1.0 cm, ensuring optimal adaptation and stability within the metatarsal osteotomy gap.

#### 2.1.2. Metatarsal Lengthening and Fixation

After preparing the calcaneal bone graft, a longitudinal incision was made in the third and fourth intermetatarsal space, and a base osteotomy was performed. The calcaneal bone autograft was then inserted at the base of the third and fourth metatarsals to achieve the desired elongation.

Based on the length achieved in the third and fourth metatarsals, a distal shortening osteotomy was performed on the second and fifth metatarsals to maintain forefoot balance. The fragments were stabilized using four 1.6 mm K-wires, inserted from the distal phalanx to the base of the lesser metatarsals.

Sutures were performed using 2/0 synthetic absorbable sutures for the closure of the capsules of the first MTP and lesser rays, 3/0 sutures for the closure of the fascia, and 4/0 Biosyn^TM^ monofilament sutures for continuous skin closure in all incisions.

### 2.2. Postsurgical Procedure and Evolution

The patient underwent postsurgical immobilization with a plaster cast. It was at week 8, after radiographic review, when the K-wire fixation of the second, fourth, and fifth radii was removed. At week 10, K-wire fixation of the third radius was removed, and partial loading began with the use of a Walker boot for 4 weeks.

The decision to remove the K-wire fixation of the third metatarsal at week 10, instead of week 8, was based on radiographic follow-up, which indicated that additional stabilization time was necessary to ensure proper bone consolidation. This approach aimed to minimize the risk of delayed healing or complications associated with premature removal.

After 16 weeks, the patient had no pain or limitations and started to wear physiological footwear. At 6 months, she began to practice running and the metatarsal adequately accepted the ground reaction force.

## 3. Results

Post-surgical radiological images show a good position and alignment of the metatarsal parabola. We can observe the favourable evolution of bone consolidation in the shortening osteotomies and the correct integration of the bone autograft. Additionally, a bone defect is visible at the lateral aspect of the calcaneus after removal of the autogenous graft. This defect will progressively fill with new bone over the following months until a normal calcaneal morphology is restored.

**Figure 3:**
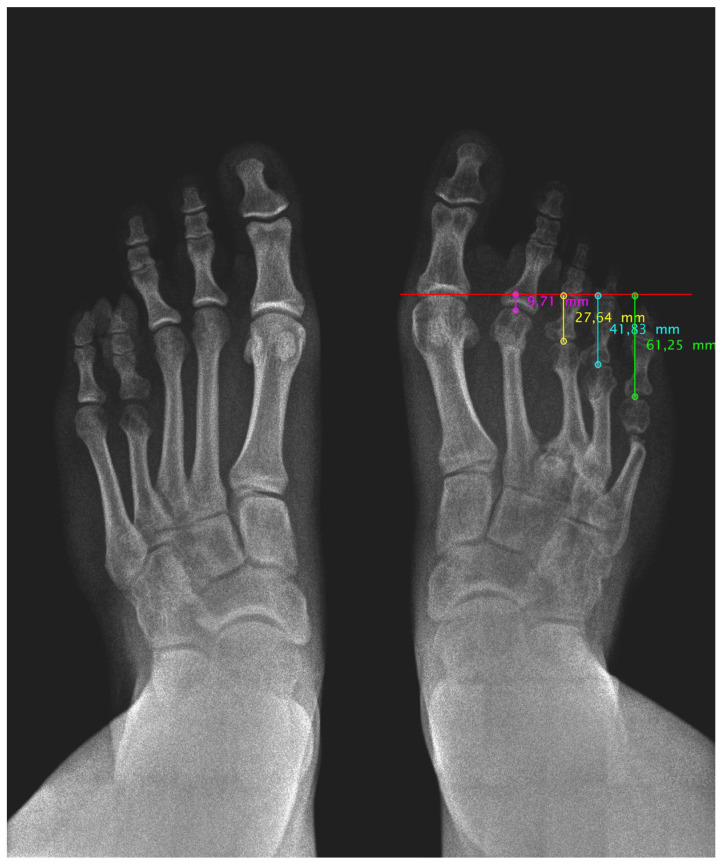
Radiological image 24 months post surgery of both feet in loading. Improvement of the metatarsal parabola harmony can be seen.

Anteroposterior and lateral radiographs were taken at 6, 12, 18, and 24 months post surgery (Figure 3). The radiological study shows correct bone consolidation, good alignment of the metatarsal parabola, as well as the remodelling process of the calcaneus following autograft removal. However, a painless non-union of the fifth metatarsal osteotomy is observed, which remains asymptomatic and does not interfere with the patient’s functionality.

**Figure 4:**
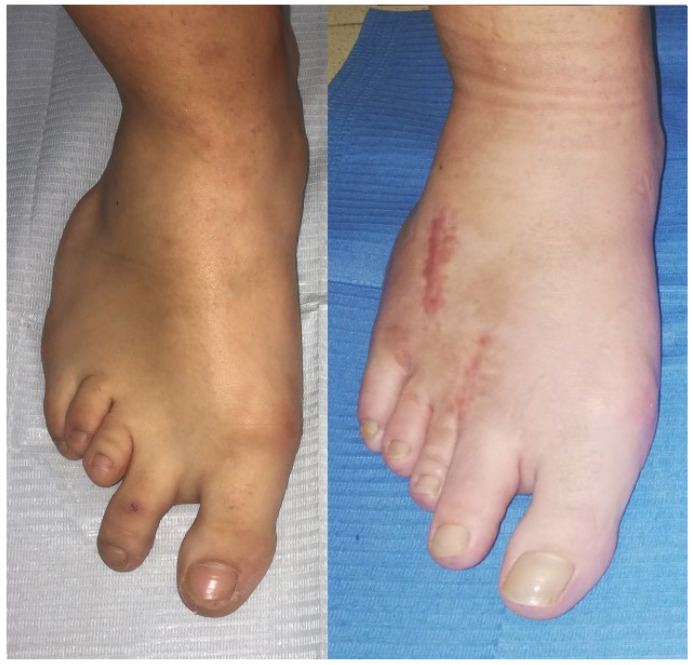
Image of the patient’s foot, shown before and after surgery to create digital harmony and improved load distribution.

There is a significant improvement in the aesthetic appearance of the foot (Figure 4). Although a slight longitudinal difference persists, the overall alignment is excellent. However, the primary focus has never been the cosmetic outcome, but rather the functional aspect. After two years of follow-up, our patient maintains a good quality of life, without pain, which remains our main goal.

## 4. Discussion

Surgical treatment of brachymetatarsia is primarily aimed at restoring the length of the affected metatarsal to improve the function and aesthetics of the foot. The most commonly used techniques include one-stage lengthening, usually involving bone grafting, and gradual distraction by osteogenesis. Both options have advantages and limitations that have been extensively described in the literature.

### 4.1. One-Stage Elongation

One-stage lengthening is often performed by the interposition of autologous, allogeneic, or synthetic bone grafts. This technique allows for the correction of moderate deformities with an average length gain of 14.4 mm [17]. However, the technique has limitations related to soft tissue tension, which restricts lengthening to less than 15 mm in most cases to avoid complications such as ischaemia or skin necrosis [18,19].

Giannini et al. achieved good results using homologous bone grafts in 41 feet, with an average gain of 13 mm and complete bone healing in all cases, with no significant complications [18]. Waizy et al. reported the use of autologous fibula grafts fixed with locking plates in eight patients, obtaining an average elongation of 9.01 mm without postoperative complications [20]. However, the use of autologous grafts is associated with donor site morbidity, whereas allogeneic grafts have lower osteogenic capacity and a higher risk of resorption [21].

### 4.2. Gradual Distraction

The gradual distraction technique, performed with external fixators, is an alternative that allows elongations greater than 15 mm due to its ability to lengthen both bone and soft tissues progressively. Recent studies show an average gain of 17.2 mm and an elongation rate of 37% [22]. However, this technique is associated with a higher incidence of complications (36.5%) compared to one-stage lengthening (21.1%), with infections, fractures, and joint subluxations being the most frequent [23,24].

The healing rate in gradual distraction (61.4 days/cm) is significantly higher than in one-stage procedures, reflecting a longer recovery time for patients [18]. In addition, the use of circular devices has demonstrated a reduction in the incidence of joint misalignments and subluxations compared with monolateral fixators, although their use remains limited [8,16,24].

### 4.3. Alternative and Combined Techniques

Z-osteotomy is a technique that allows lengthening without the need for bone grafting, which reduces morbidity at the donor site. However, it is limited to minor elongations and is not applicable in all cases [23]. On the other hand, some authors have combined gradual distraction with bone graft interposition to optimize results and reduce the duration of the distraction procedure, obtaining good results without significant complications [22].

### 4.4. Use of Calcaneal Bone Graft in Metatarsal Lengthening

The calcaneus has been widely recognized as a valuable donor site for bone grafting in foot and ankle surgery due to its high cancellous bone content and osteogenic potential [25]. Its use in metatarsal lengthening procedures provides structural support while promoting rapid integration and remodeling [26]. Studies have demonstrated that calcaneal grafts maintain their volume and structural integrity over time, reducing the risk of resorption seen with other grafting techniques [8]. Furthermore, harvesting from the lateral calcaneus minimizes donor site morbidity while preserving the medial cortical wall, which is crucial for maintaining stability and avoiding fractures [27]. Ortiz et al. highlighted the advantages of calcaneal grafts in reconstructive foot surgery, emphasizing their excellent vascularization and ability to facilitate early weight-bearing [25].

### 4.5. Limitations

This study has several important limitations. First, most of the studies reviewed are retrospective in design and lack randomized controlled trials, which limits the robustness of the conclusions. In addition, heterogeneity in surgical techniques and functional assessment tools makes direct comparison of results difficult. Small sample sizes and limited follow-up in some studies also restrict the ability to assess long-term complications. Finally, our results are based solely on a subjective assessment proposed by the authors, which could introduce biases in the interpretation of the data.

## 5. Conclusions

Gradual distraction, especially when combined with techniques such as osteotomy or metatarsal reaming, appears to be the safest option for cases requiring significant lengthening, either more than 40% of the original metatarsal length or more than 15mm. This technique allows progressive control of lengthening, reduces the risk of neurovascular complications and facilitates soft tissue adaptation. In addition, the use of a K-wire after removal of the distraction device helps to stabilize the metatarsal, ensuring correct alignment.

In our case, we chose to avoid excessive lengthening and prioritized a single-stage technique, using a calcaneal allograft in combination with reduction in the adjacent metatarsals. Although this approach required additional surgical procedures, we felt that it offered our patient a more controlled postoperative period, with lower risks of infection and associated complications. Despite the need to maintain a period of unsupported immobilization for eight weeks, the results were satisfactory in terms of function and aesthetics.

## Data Availability

Data are contained within the article.
